
JOURNAL INFORMATION
==============================
NLM Title Abbreviation: Wirtschaftsdienst
Journal ID: Wirtschaftsdienst
NLM Journal ID: 101086612

Wirtschaftsdienst (Hamburg, Germany : 1949)

ISSN: 0043-6275

ARTICLE INFORMATION
==============================
PMCID: PMC8684781
PMID: 34955566
DOI: 10.1007/s10273-021-3075-2
Article ID: 3075
Article version: 1
Subjects: Kurz Kommentiert

Deglobalisierung: Wird die Inflation angeheizt?

Deglobalisation: Is inflation being fuelled?

Marin Dalia dalia.marin@tum.de

grid.6936.a 0000000123222966 TUM School of Management, Technische Universität München, Arcisstrasse 21, 80333 München, Deutschland
Electronic publication date: 2021 Dec 19
Print publication date: 2021
Volume: 101
Issue: 12
First page: 924
Last page: 924
Copyright: © Der/die Autor:in 2021
License: Open Access: Dieser Artikel wird unter der Creative Commons Namensnennung 4.0 International Lizenz veröffentlicht (https://creativecommons.org/licenses/by/4.0/deed.de).
Open Access wird durch die ZBW — Leibniz-Informationszentrum Wirtschaft gefördert.
License URL: https://creativecommons.org/licenses/by/4.0/

issue-copyright-statement: © The Author(s) 2021

==============================

DOI: 10.1007/s10273-021-3075-2

Deglobalisierung

### Wird die Inflation angeheizt?

Inflation scheint heutzutage in aller Munde zu sein. Die Debatte dreht sich in der Regel darum, ob die massiven geld- und fiskalpolitischen Anreize der USA die Inflationserwartungen beflügeln und die Preise außer Kontrolle geraten lassen werden. Aber es gibt noch einen anderen Trend, der ebenfalls einen Inflationsdruck erzeugen könnte: die Deglobalisierung. Seit der globalen Finanzkrise 2008 findet eine Deglobalisierung statt und die Corona-Pandemie hat diesen Trend erheblich beschleunigt. Anhand von Daten aus der Finanzkrise prognostizieren Kemal Kilic und ich, dass der COVID-19-Schock wahrscheinlich zu einem Rückgang der grenzüberschreitenden Wertschöpfungsketten um 35 % führen wird – dem Hauptfaktor der Globalisierung in den vergangenen drei Jahrzehnten.

Eine kürzlich durchgeführte Umfrage des Münchner ifo-Instituts stützt diese Schlussfolgerung. Die Studie ergab, dass etwa 19 % der deutschen Unternehmen des Verarbeitenden Gewerbes planen, ihre Produktion wieder nach Deutschland zu verlagern. 12 % davon planen, Vorleistungen von deutschen Zulieferern zu beziehen und 7 % selbst zu produzieren. Steigende Transportkosten werden die Abkehr von globalen Wertschöpfungsketten wahrscheinlich noch beschleunigen. Während der Pandemie haben sich die Kosten für Container, die für den Warentransport von Asien nach Europa und in die USA verwendet werden, fast verzehnfacht, und die Beschäftigten der Branche, die mit immer härteren Arbeitsbedingungen konfrontiert sind, verlassen ihre Arbeitsplätze.

Diese Entwicklungen haben die Rentabilität globaler Wertschöpfungsketten erheblich geschmälert. Die Unternehmen nutzten die Auslandsverlagerung, um von den viel niedrigeren Löhnen im postkommunistischen Europa und in China zu profitieren, insbesondere nach dem Beitritt Chinas zur Welthandelsorganisation 2001. Und eine Revolution im Transportsektor – die Containerisierung – erleichterte diesen Prozess, indem sie dazu beitrug, die Transportkosten so niedrig zu halten, dass sie die Lohnunterschiede nicht ausglichen. Heute sind diese Unterschiede kleiner und die Transportkosten sind viel höher, was den Anreiz für die Unternehmen schwächt, ihre Aktivitäten an weit entfernten Standorten zu halten. Darüber hinaus verringert Onshoring (oder Nearshoring) ihre Anfälligkeit für globale Schocks. Charles Goodhart und Manoj Pradhan zufolge ist der daraus resultierende Rückzug aus der Globalisierung, verbunden mit der Alterung der Bevölkerung in China und den reichen Volkswirtschaften, ein Rezept für Inflation. Ihrer Ansicht nach hat die Globalisierung die Preise drei Jahrzehnte lang niedrig gehalten: Als die Produktion in Niedriglohnländer verlagert wurde, wurden die Löhne gedrückt. Da es immer schwieriger wird, billige Arbeitskräfte zu finden, ob im In- oder Ausland, wird die Verhandlungsmacht der Beschäftigten in den Industrieländern zunehmen, was den Inflationsdruck verstärkt.

Haben sie Recht? Werden die Inflation der Verbraucherpreise und die Löhne nach der Pandemie anziehen, wenn die Welt in eine neue Ära der Deglobalisierung eintritt? Die Antwort hängt davon ab, inwieweit der Umbruch im Verkehrssektor anhält. Wenn der Sektor, wie einige behaupten, einen grundlegenden Wandel durchläuft, bei dem die Kosten hoch bleiben, könnte dies in den reichen Ländern zu einer Lohn-Preis-Spirale führen, da die Arbeitnehmer:innen versuchen, einen Ausgleich für die steigenden Preise zu erhalten. Wenn die Unternehmen jedoch ihre Aktivitäten wieder ins Inland verlagern, werden die Auswirkungen der höheren Transportkosten deutlich abgeschwächt. Außerdem dürfte das Argument, dass der Lohndruck die Inflation anheizen wird, nicht sehr stichhaltig sein. Schließlich können Unternehmen in Hochlohnländern in vielen Fällen verstärkt Roboter einsetzen, anstatt teurere lokale Arbeitskräfte einzustellen. Und unsere Untersuchungen deuten tatsächlich darauf hin, dass die Verlagerung von Lieferketten den Einsatz von Robotern in den Industrieländern fördert.

Die Robotisierung wird auch die Auswirkungen der demografischen Entwicklung auf die Löhne verringern. Die Unternehmen haben die Alterung der Bevölkerung – und die damit verbundene Schrumpfung der Erwerbstätigenzahlen – seit den 1990er Jahren vorausgesehen. Und wie Daron Acemoglu und Pascual Restrepo zeigen, haben Länder, in denen die Bevölkerung schneller altert, auch schneller Roboter eingeführt. In Deutschland, einer der am schnellsten alternden Gesellschaften der Welt, stieg die Zahl der Roboter pro 1.000 Angestellte von unter zwei Mitte 1990 auf vier 2019. Die Robotisierung wird den Arbeitskräftemangel nicht nur abmildern, sie könnte ihn sogar ausgleichen und zu einem Arbeitskräfteüberschuss führen. Wie Acemoglu und Restrepo hervorgehoben haben, hat die Automatisierung in den vergangenen drei Jahrzehnten weit mehr Arbeitnehmer:innen verdrängt als neue Arbeitsplätze geschaffen. Dies birgt zwar sicherlich Risiken für die Angestellten, insbesondere für die, die in den fortgeschrittenen Volkswirtschaften mit steigenden Preisen konfrontiert sind, aber es deutet auch darauf hin, dass die Deglobalisierung in absehbarer Zeit nicht zu einem Inflationsanstieg führen wird.

Dalia Marin
TUM School of Management München
dalia.marin@tum.de

© Der/die Autor:in 2021. Open Access: Dieser Artikel wird unter der Creative Commons Namensnennung 4.0 International Lizenz veröffentlicht (creativecommons.org/licenses/by/4.0/deed.de).

Open Access wird durch die ZBW – Leibniz-Informationszentrum Wirtschaft gefördert.
